# Association of institutional transition of cancer care with mortality in elderly patients with lung cancer: a retrospective cohort study using national claim data

**DOI:** 10.1186/s12885-022-09590-5

**Published:** 2022-04-25

**Authors:** Kyu-Tae Han, Jongwha Chang, Dong-Woo Choi, Seungju Kim, Dong Jun Kim, Yoon-Jung Chang, Sun Jung Kim

**Affiliations:** 1 Division of Cancer Control & Policy, National Cancer Control Institute, National Cancer Center, Goyang, Republic of Korea; 2 Department of Healthcare Administration, College of Business, Texas Woman’s University, Denton, TX USA; 3 Cancer Big Data Center, National Cancer Control Institute, National Cancer Center, Goyang, Republic of Korea; 4 Department of Nursing, College of Nursing, The Catholic University of Korea, Seoul, Republic of Korea; 5 Department of Health Administration and Management, College of Medical Science, Soonchunhyang University, 22 Soonchunhyang-ro, Asan, 31538 Republic of Korea; 6 Center for Healthcare Management Science, Soonchunhyang University, Asan, Republic of Korea; 7 Department of Software Convergence, Soonchunhyang University, Asan, Republic of Korea

**Keywords:** Institutional transition of cancer care, Lung Cancer, Cox proportional hazard model

## Abstract

**Background:**

Although survival based outcomes of lung cancer patients have been well developed, institutional transition of cancer care, that is, when patients transfer from primary visiting hospitals to other hospitals, and mortality have not yet been explored using a large-scale representative population-based sample.

**Methods:**

Data from the Korean National Elderly Sampled Cohort survey were used to identify patients with lung cancer who were diagnosed during 2005–2013 and followed up with for at least 1 year after diagnosis (3738 patients with lung cancer aged over 60 years). First, the authors examined the distribution of the study population by mortality, and Kaplan-Meier survival curves/log-rank test were used to compare mortality based on institutional transition of cancer care. Survival analysis using the Cox proportional hazard model was conducted after controlling for all other variables.

**Results:**

Results showed that 1-year mortality was higher in patients who underwent institutional transition of cancer care during 30 days after diagnosis (44.2% vs. 39.7%, *p* = .027); however, this was not associated with 5-year mortality. The Cox proportional hazard model showed that patients who underwent institutional transition of cancer care during 30 days after diagnosis exhibited statistically significant associations with high mortality for 1 year and 5 years (1-year mortality, Hazard ratio [HR]: 1.279, *p* = .001; 5-year mortality, HR: 1.158, *p* = .002).

**Conclusion:**

This study found that institutional transition of cancer care was associated with higher mortality among elderly patients with lung cancer. Future consideration should also be given to the limitation of patients’ choice when opting for institutional transition of care since there are currently no control mechanisms in this regard. Results of this study merit health policymakers’ attention.

## Background

Cancer is a significant cause of death worldwide, and it continues to be one of the most important modern public health concerns [1]. Over the past decades, cancer has been the leading cause of death in South Korea. In 2018, there were 28,628 lung cancer cases and 17,853 deaths in South Korea. Of 243,837 cancer cases, 53% were male, and of 79,153 cancer related deaths, 74% were male [2]. Age-standardized mortality rate in lung cancer was 15.7 per 100,000 individuals in 2018, making it the leading cause of cancer-related deaths [3]. In fact, both men and women aged 60 years or above have been projected to have the highest mortality rates resulting from lung cancer in 2020 [4]. As South Korea becomes an aged society, cancer incidents and deaths will increase, making it imperative to systematically manage it and identify factors associated with patient survival [5, 6]. Lung cancer occurs predominantly in the elderly group, with older individuals displaying poor prognosis. Several factors such as age, sex, socio-economic status, lung function, clinical and pathological stage, body constitution, comorbidity, malnourishment, optimal treatment, and—most importantly—a history of tobacco use influencing the survival of lung cancer patients [7–9].

The transition of care for patients requires a change of healthcare providers responsible for managing the care of a patient [10]. This transition of caregivers makes for a vulnerable situation which may lead to lapses in quality and safety [11]. Transitions of care are associated with a high rate of complications, particularly for elderly people with chronic health conditions, potentially resulting in adverse events and high health care costs [12–14]. In South Korea, the National Health Insurance (NHI) was introduced in 2000, strengthening the insurance coverage for cancer, including reducing copayment in 2005, which was expanded in 2009. Consequently, cancer patients in South Korean can now visit medical institutions of their choice and receive cancer diagnosis and treatment by paying only 5% of the total medical cost. However, there is no strict gatekeeping system for controlling healthcare utilization, and it is relatively easy for patients to access primary and secondary care as well as services in tertiary hospitals [15]. Although oncologists or other physicians may refer patients to other hospitals, in South Korea, patients’ intention to visit superior or mega-sized hospitals drives institutional transition of cancer care. Thus, lack of mechanisms that guide patients’ choice for optimal cancer care and the absence of a gatekeeping system can lead to quality issues and inefficiency in healthcare delivery [16].

Previous research has reported that continuity of care is a core element of medical care and represents a trustful and responsible therapeutic relationship between patients and their care providers [17–19]. Additionally, a long-term, sustained, and trusting relationship between physicians and patients can enhance mutual communication and effective disease management, which in turn improve patient outcomes, particularly for chronic diseases [20, 21]. Furthermore, continuity of care has been reported to reduce mortality among various cancer types [22–26]. However, institutional transition of cancer care and its association with mortality has not been studied yet. Continuity of care measures the number of physicians providing service to one patient and the percentage of care provided by each physician. Since the NHI data do not include individual physician records but institutional level information, measuring institutional transition of cancer care and its outcomes is more appropriate. To the best of the authors’ knowledge, no study has yet examined the association between institutional transition of cancer care and mortality of elderly lung cancer patients. Therefore, the purpose of this study was to investigate whether institutional transition of cancer care is associated with mortality within 1 year and 5 years for elderly lung cancer patients using Korean National Elderly Sampled Cohort data, by means of end-of-life healthcare utilization after policy implementation.

## Methods

### Study population

The Korean National Elderly Sampled Cohort study collected data from 10% (5.5 million people aged over 60 years) of the Korean elderly population who were patients in 2002. The study used a random sampling technique; follow-ups were conducted from 2002 through 2015 (*n* = 550,000). The data included patient information including demographic and socio-economic characteristics, healthcare utilization and treatment details, and medical institution characteristics.

**Fig. 1:**
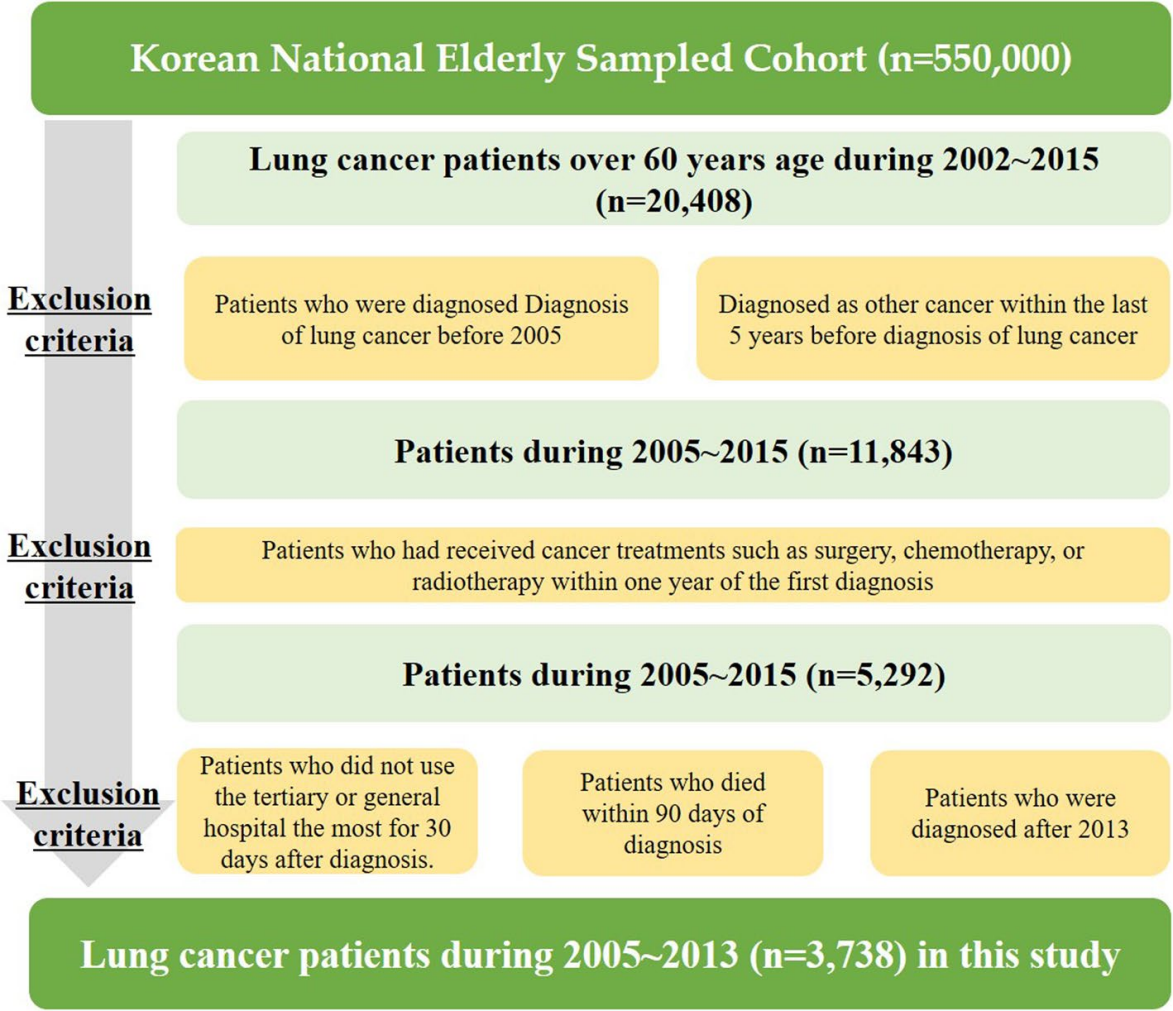
Selection of study population to investigate the relationship between institutional transition and mortality in cancer patients

To investigate the association between institutional transition of cancer care and survival, we only included cancer patients who were diagnosed with lung cancer (International Classification of Diseases [ICD]-10: C34). Thus, those who were newly diagnosed before 2005 or had been diagnosed with other forms of cancer in the preceding 5 years before lung cancer were excluded from this study. Additionally, only those who had received cancer treatments such as surgery, chemotherapy, or radiotherapy within 1 year of the first diagnosis were included in this study. Further, only those patients who visited tertiary or general hospitals during the 30 days after diagnosis were included. The preceding two criteria were used to ensure homogeneity of patients. The authors excluded patients observed for less than 90 days after diagnosis to reduce immortal time bias. Only cancer patients who were diagnosed during 2005–2013 were included in the follow up for at least 1 year after diagnosis. Consequently, the data used in this study consisted of 3738 lung cancer patients aged over 60 years (Fig. 1).

### Variables

The authors defined the first date of visiting hospital, either inpatient or outpatient care, due to major diagnosis of lung cancer as the index date, and each patient was traced for a minimum of 1 year (365 days) or maximum of 5 years (1825 days). Patients who died within 1 year or 5 years were classified within the Died group, regardless of the cause of death, and those who lived were classified within the Survived group.

The primary variable of interest to examine the association between institutional transition of cancer care and mortality was the institutional transition from the hospital most visited by the patient within 90 days of being diagnosed. First, the authors summarized the medical costs of each general hospital visited by the patient within 30 days of being diagnosed regardless of whether the patient received treatment at each visit. The hospital where the patient incurred the highest medical expenses was defined as the primarily treatment hospital for a patient. Subsequently, the authors similarly defined the most visited hospital during 31–90 days after the index date. Considering the government guideline for optimal time-to-treatment as 30 days after diagnosis of cancer and the result of a previous study, if patients changed their primary treatment hospital within 31–90 days, after 30 days of diagnosis, the authors categorized them under the Changed group [ 27].

The other independent variables included in this study were sex, age, types of insurance coverage, economic status, area of residence (capital area, metropolitan, or rural), Charlson Comorbidity Index (CCI), year of diagnosis, types of treatment within 1 year, and the size or location of the major general hospital with the highest medical expenses within 1 month after diagnosis. Regarding classification of the Korean NHI, 97% of individuals were NHI beneficiaries and were classified into the NHI employee or self-employed groups. The NHI employee group included all employees and employers, with their household members also being covered. The NHI self-employed group included all other individuals, with insurance premiums being calculated based on income and property. Furthermore, the Medical-aid group included 3% of low-income or individuals with disabilities who do not pay insurance premium. Generally, NHI beneficiaries only pay 5% copayment for medical cost due to cancer care, and the Medical-aid group pays 0% of inpatient care and 0–5% of outpatient care. Economic status was calculated based on the insurance premium paid individuals depending on their economic level, and was classified as ~ 20 (low), 31 to 50 (mid-low), 51 to 80 (mid-high), and 81+ (high) percentiles. The CCI was utilized to incorporate clinical severity, calculated based on medical and symptom records after cancer diagnosis and excluded the score due to cancer; the CCI was classified into 0–2, 3–4, or ≥ 5. The types of treatment within 1 year were defined based on whether patients were provided treatment such as surgery, chemotherapy, or radiotherapy for the lung cancer. Subsequently, the authors classified patients into three categories, namely “Surgery and Chemotherapy or Radiotherapy,” “Only Surgery,” and “Chemotherapy or Radiotherapy.” The size of the hospital (≤ 700 beds, 701–1000 beds, 1001–1500 beds, or ≥ 1501 beds) and the location (capital area, metropolitan, or rural) of the general hospital that was primarily visited for 1 month after diagnosis was defined based on characteristics of the general hospital where the patient had the highest expenditure within 1 month of cancer diagnosis.

### Statistical analysis

The authors examined the distribution of the study population by mortality and conducted chi-square tests to compare categorical independent variables. Additionally, the authors analyzed the mean and standard deviation for the continuous variables such as age by mortality. Then, the authors used the Kaplan-Meier survival curves and log-rank test to compare mortality based on institutional transition of cancer care. Survival analysis using the Cox proportional hazard model was conducted after controlling for independent variables such as sex, age, types of insurance coverage, economic status, area of residence, CCI, year of diagnosis, types of treatment, and the size or location of the major general hospital where the patient had the highest medical expenditure to investigate the association between institutional transition of cancer care and mortality by 1 year or 5 years. Further, the authors conducted sub-group survival analysis using economic status, residence area, or types of treatment to compare differences between groups. All statistical analyses were performed using the SAS statistical software version 9.4 (Cary, NC).

## Results

This study included 3738 elderly cancer patients who were diagnosed with lung cancer and received treatment. Table 1 shows the distribution of the study population by 1-year or 5-year mortality. Results show that 40.6% or 76.2% patients died within 1 year or 5 years after the first diagnosis of lung cancer, respectively. The 1-year mortality was higher in patients who underwent institutional transition of cancer care within 30 days of being diagnosed (44.2% vs. 39.7%, *p* = .027), but it was not associated with 5-year mortality. As per sex and age, more male patients and older patients died in both the 1-year and 5-year mortality groups (*p* < .001). Lower economic status of patients was not associated with 1-year mortality, but it had a statistically significant association with 5-year mortality (*p* < .001). Patients who lived in the capital area had lower rates of mortality in both outcome variables as compared to those living in other regions, and year of diagnosis was inversely associated with mortality (*p* < .005). With regard to type of treatment received, patients who had received chemotherapy or radiotherapy had higher mortality rates than those who had undergone surgery (*p* < .001). Based on the characteristics of the hospital primarily visited within 30 days, smaller hospitals or hospitals which were located in non-capital areas reported higher death rates (*p* < .001).

**Table 1:** Lung cancer patients’ characteristics by 1-year and 5-year mortality status

Variables	Total		1-year mortality			5-year mortality		
			Death	Survived	***P***-value	Death	Survived	***P***-value
	N	%	%	%		%	%	
**Institutional transition of cancer care (1 month Vs. 2–3 months)**								
Changed	738	19.7	44.2	55.8	0.027	75.5	24.5	0.610
Unchanged	3000	80.3	39.7	60.3		76.4	23.6	
**Sex**								
Male	2814	75.3	43.0	57.0	<.001	79.2	20.8	<.001
Female	924	24.7	33.1	66.9		66.9	33.1	
**Age (Years)**^**a**^	72.9	5.0	5.3	4.8	<.001	5.2	4.4	<.001
**Types of insurance coverage**								
Medical-aid	341	9.1	40.8	59.2	0.792	76.8	23.2	0.507
NHI, Self-employed	1136	30.4	41.4	58.6		77.3	22.7	
NHI, Employee	2261	60.5	40.2	59.8		75.5	24.5	
**Economic status**								
Low	784	21.0	41.8	58.2	0.415	77.9	22.1	<.001
Mid-low	645	17.3	42.8	57.2		80.8	19.2	
Mid-high	943	25.2	39.6	60.4		76.7	23.3	
High	1366	36.5	39.5	60.5		72.7	27.3	
**Residence area**								
Capital area	1436	38.4	37.6	62.4	0.012	74.0	26.0	0.025
Metropolitan	774	20.7	43.3	56.7		78.8	21.2	
Rural	1528	40.9	42.0	58.0		77.0	23.0	
**Charlson Comorbidity Index**								
~ 2	1799	48.1	38.9	61.1	0.055	75.2	24.8	0.398
3 ~ 4	1189	31.8	41.0	59.0		77.1	22.9	
5 ~	750	20.1	44.0	56.0		77.1	22.9	
**Year of diagnosis**								
2005	402	10.8	46.3	53.7	0.017	79.6	20.4	<.001
2006	409	10.9	34.0	66.0		78.0	22.0	
2007	437	11.7	41.4	58.6		78.3	21.7	
2008	407	10.9	39.8	60.2		78.6	21.4	
2009	400	10.7	39.5	60.5		78.8	21.3	
2010	464	12.4	45.5	54.5		82.1	17.9	
2011	403	10.8	40.2	59.8		76.9	23.1	
2012	412	11.0	38.8	61.2		69.7	30.3	
2013	404	10.8	39.1	60.9		62.9	37.1	
**Types of treatment after diagnosis**								
Surgery & Chemotherapy or Radiotherapy	581	15.5	29.8	70.2	<.001	66.8	33.2	<.001
Only Surgery	790	21.1	17.3	82.7		40.8	59.2	
Chemotherapy or Radiotherapy	2367	63.3	51.0	49.0		90.3	9.7	
**The size of a general hospital mainly visited 1 month after diagnosis**								
General hospital (> 1501 beds)	924	24.7	31.8	68.2	<.001	68.6	31.4	<.001
General hospital (1001 ~ 1500 beds)	1019	27.3	40.0	60.0		75.8	24.2	
General hospital (701 ~ 1000 beds)	1215	32.5	46.0	54.0		80.8	19.2	
General hospital (≤700 beds)	580	15.5	44.1	55.9		79.3	20.7	
**The location of a general hospital mainly visited 1 month after diagnosis**								
Capital area	2093	56.0	36.5	63.5	<.001	72.2	27.8	<.001
Metropolitan	959	25.7	43.8	56.2		80.5	19.5	
Rural	686	18.4	48.7	51.3		82.4	17.6	
**Total**	**3738**	**100.0**	**40.6**	**59.4**		**76.2**	**23.8**	

^a^The mean or standard deviation for age and the results of analysis of variance

**Fig. 2:**
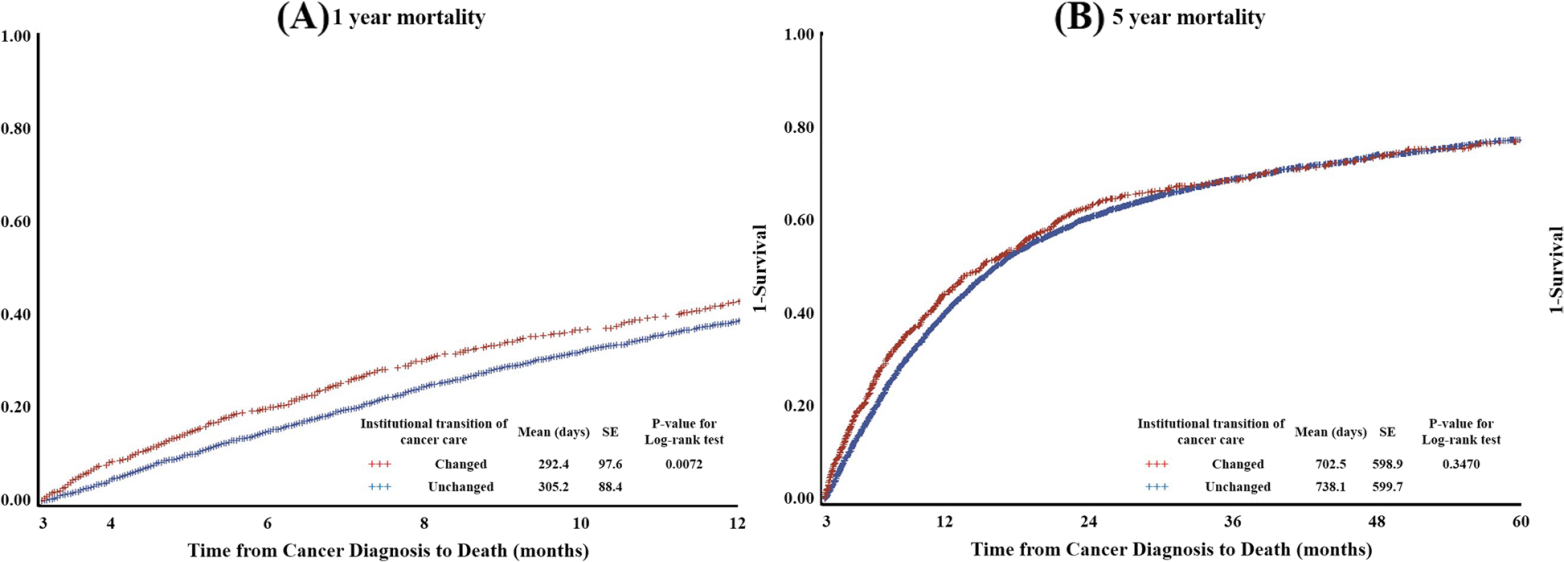
Kaplan-Meier Survival Curves by the institutional transition of cancer care and mortality

Figure 2 shows the Kaplan-Meier survival curves and log-rank test results. Patients who underwent institutional transition of cancer care between 31 and 90 days, compared to those who did so within 30 days after the first diagnosis, had a shorter survival period than patients who did not (1-year survival period; changed, Mean and SD: 292.4 and 97.6; unchanged, Mean and SD: 305.2 and 88.4; *P*-value for log-rank test of 1-year mortality: 0.007). However, the difference in survival period was not statistically significant in the 5-year mortality group (5-year survival period; changed, Mean and SD: 702.5 and 598.9; unchanged, Mean and SD: 738.1 and 599.7; *P*-value for log-rank test of 5-year mortality: 0.347).

Table 2 shows the results of survival analysis using the Cox proportional hazard model that was used to investigate the association of variables with 1-year or 5-year mortality. Institutional transition of cancer care within 30 days of the first diagnosis was significantly associated with high mortality for 1 year and 5 years (1-year mortality, Hazard ratio [HR]: 1.279, *p* = .001; 5-year mortality, HR: 1.158, *p* = .002). Men were at a greater risk of death than women. The age of patients was associated with higher mortality. Economic status of patients was not associated with higher risk in the 1-year mortality group, but patients with a lower economic level had higher risk in the 5-year mortality group (Low, HR: 1.136, *p* = .046; Mid-low, HR: 1.149, *p* = .015; Mid-high, HR: 1.023, *p* = .611). Patients with a CCI score of higher than 5 were at a greater risk of death, for both 1 year and 5 years, as compared to patients with a CCI score of lesser than 2. The year of diagnosis was inversely associated with risk of death. Based on the type of treatment received, patients who received only surgery or surgery and chemotherapy or radiotherapy were at a lower risk of death than patients who received chemotherapy or radiotherapy and not surgical treatment. The hospital primarily visited during 30 days after the first diagnosis was associated with risk of death; patients who visited a hospital with more than 1501 beds had a lower risk for 1-year and 5-year mortality than those who visited a hospital with less than 700 beds (1-year mortality, HR: 0.739, *p* < .001; 5-year mortality, HR: 0.774, *p* < .001). Additionally, those who visited hospitals with 1001 to 1500 beds had low risk for 5-year mortality (HR: 0.818, *p* = .001). Based on the hospital location, the risk of death of patients in the capital area was lower than that of those in rural areas (1-year mortality, HR: 0.835, *p* = .038; 5-year mortality, HR: 0.844, *p* = .008).

**Table 2:** Association between institutional transition of cancer care and mortality after adjusting for other covariates

Variables	1-year mortality				5-year mortality			
	HR	95% CI		***P***-value	HR	95% CI		***P***-value
**Institutional transition of cancer care (1 month Vs. 2**–**3 months)**								
Changed	1.279	1.129	1.449	<.001	1.158	1.054	1.273	0.002
Unchanged	1.000	–	–	–	1.000	–	–	–
**Sex**								
Male	1.318	1.159	1.497	<.001	1.318	1.203	1.443	<.001
Female	1.000	–	–	–	1.000	–	–	–
**Age (Years)**	1.041	1.030	1.052	<.001	1.037	1.029	1.045	<.001
**Types of insurance coverage**								
Medical-aid	0.909	0.722	1.144	0.415	0.903	0.763	1.067	0.230
NHI, Self-employed	1.030	0.916	1.159	0.621	0.996	0.914	1.085	0.921
NHI, Employee	1.000	–	–	–	1.000	–	–	–
**Economic status**								
Low	1.038	0.874	1.233	0.668	1.136	1.002	1.288	0.046
Mid-low	1.026	0.880	1.197	0.739	1.149	1.027	1.285	0.015
Mid-high	0.940	0.822	1.074	0.361	1.026	0.931	1.130	0.611
High	1.000	–	–	–	1.000	–	–	–
**Residence area**								
Capital area	1.000	–	–	–	1.000	–	–	–
Metropolitan	1.129	0.944	1.349	0.185	1.046	0.919	1.191	0.494
Rural	1.047	0.905	1.211	0.537	1.006	0.907	1.115	0.913
**Charlson Comorbidity Index**								
~ 2	1.000	–	–	–	1.000	–	–	–
3 ~ 5	1.009	0.898	1.134	0.882	1.056	0.970	1.149	0.209
5 ~	1.160	1.016	1.324	0.028	1.131	1.025	1.248	0.014
**Year of diagnosis**	0.974	0.954	0.995	0.017	0.975	0.960	0.991	0.002
**Types of treatment after diagnosis**								
Surgery & Chemotherapy or Radiotherapy	0.531	0.452	0.624	<.001	0.495	0.443	0.552	<.001
Only Surgery	0.272	0.227	0.325	<.001	0.227	0.202	0.256	<.001
Chemotherapy or Radiotherapy	1.000	–	–	–	1.000	–	–	–
**The size of a general hospital mainly visited 1 month after diagnosis**								
General hospital (> 1501 beds)	0.739	0.622	0.879	<.001	0.774	0.684	0.877	<.001
General hospital (1001 ~ 1500 beds)	0.850	0.721	1.001	0.052	0.818	0.725	0.924	0.001
General hospital (701 ~ 1000 beds)	0.995	0.854	1.159	0.949	0.955	0.825	1.070	0.424
General hospital (≤700 beds)	1.000	–	–	–	1.000	–	–	–
**The location of the general hospital mainly visited 1 month after diagnosis**								
Capital area	0.835	0.704	0.990	0.038	0.844	0.744	0.957	0.008
Metropolitan	0.904	0.763	1.071	0.242	0.950	0.837	1.078	0.427
Rural	1.000	–	–	–	1.000	–	–	–

**Fig. 3:**
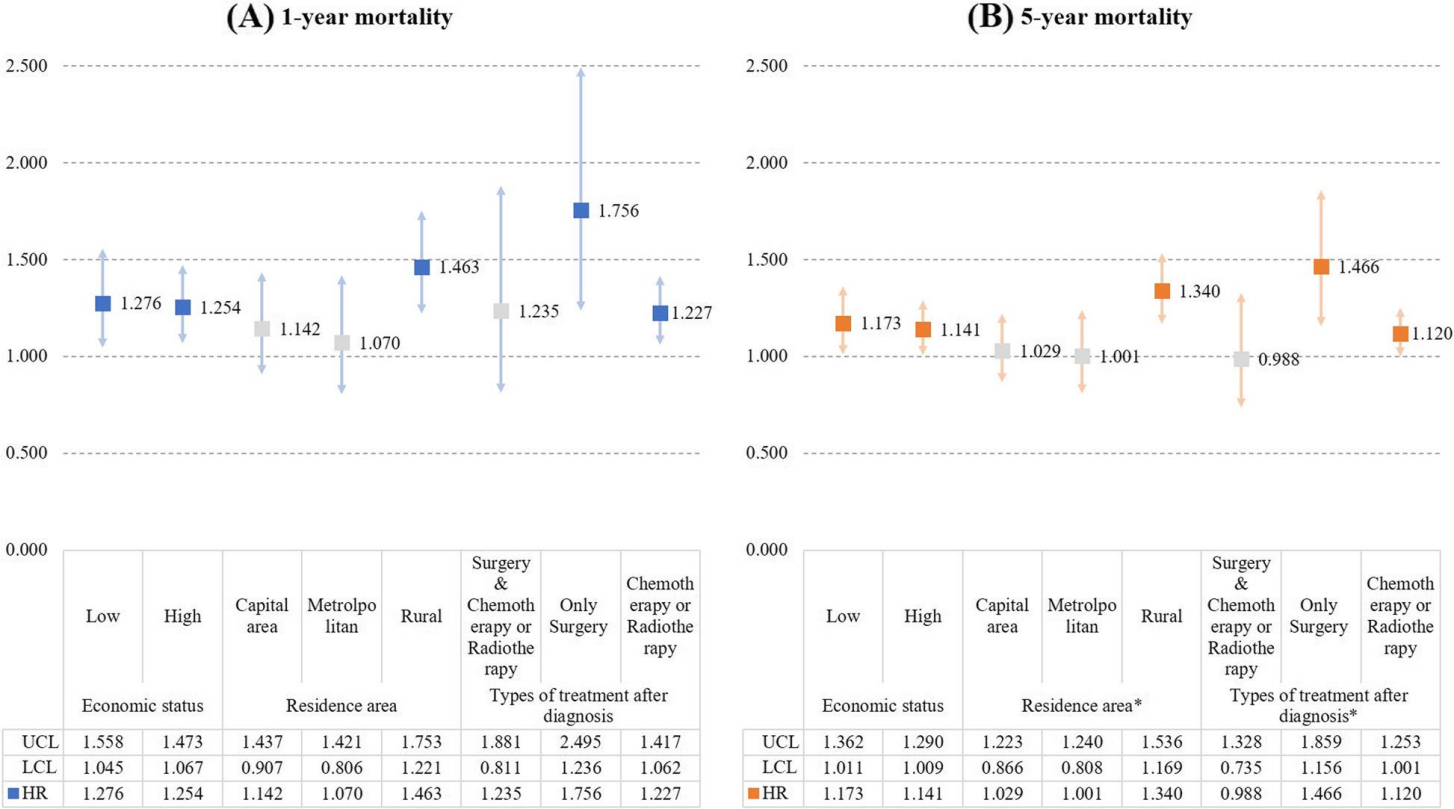
The results of sub-group Cox regression analysis according to economic status, residence area, or types of treatment. † This model was adjusted for other covariates, and the reference group was patients without institutional transition of cancer care (1 month Vs. 2–3 months). * An asterisk indicates that there is an interaction between the transition and the sub-group variable

Figure 3 shows the results of sub-group Cox regression analysis according to economic status, residence area, or types of treatment. The association between institutional transition of cancer care and mortality was greater in lower income groups and patients who lived in rural areas. Meanwhile, the risk of death by institutional transition of cancer care was greater in patients who received only surgical treatment. Regarding interactions between institutional transition of cancer care and each sub-group variable, types of treatment or residence area had statistically significant interactions with institutional transition of cancer care related to 5-year mortality.

## Discussion

In this study, the authors examined the association between institutional transition of cancer care and mortality among elderly patients with lung cancer. Using data from a large-scale Korean National Elderly Sampled Cohort, this study found evidence of higher mortality among patients with lung cancer who had experienced institutional transition of cancer care. Furthermore, the authors found that different patient characteristics such as economic status, residence area, or types of treatment were associated with mortality in the study sample.

The results of this study align with previous research findings that transition of care (discontinuity of care or fragmented care) was associated with adverse outcomes [12–14] measured by increasing mortality [22–26] among elderly lung cancer patients. One strength of our study was introducing institutional transition of cancer care in South Korea. Conventional continuity of care is not difficult to measure using health insurance claims or administrative dataset; ideas of institutional transition of care might be a helpful tool for studying other health services. Furthermore, similar to our study, a previous study explored whether patients visited multiple centers for cancer treatment [28]. However, we focused on the importance of a patient’s choice during the early phase of cancer regarding a change in treating hospital, not just on the number of hospitals visited, thus suggesting further research for facilitating patients’ informed choice and developing an optimal governmental guideline. Another strength of this study is that it used a nationwide representative sample data of elderly patients with lung cancer with a retrospective design that contributed to the robustness of this study.

The results of this study provide essential insights for the NHI program and policymakers: that is, discontinuity of lung cancer care is associated with worse patient outcomes. Under the unique healthcare system circumstances which do not limit patients’ will to transition care, it is recommended that South Korea provide incentives to health care providers to focus on patient-centered care for patients with lung cancer. Patients’ perspective on care is steadily gaining more attention as health systems worldwide aim to deliver high-quality, patient-centered care [29–31]. Especially in cancer, patient centeredness has been recognized as a top priority [32]. Increasing evidence identifies associations between patient experiences with care and clinical outcomes, including quality of life [33–36]. The key cancer care dimensions—such as high professional standard, respect, coordination of care, clear and tailored information, rapid diagnosis and treatment, and caring caregivers—need to be emphasized [37]. In addition, with regard to factors associated with patients’ choice of hospital, Korean government introduced public reporting of cancer outcomes based on a healthcare assessment by the Health Insurance Review and Assessment service (HIRA) to assist patient decision making since 2008. The data were limited since only certain outcomes, such as surgical outcomes in each hospital, were reported. Thus, policy makers and professionals related to cancer care have to consider means through which patients can make an informed choice about their treatment.

Although this study provides insights and has strengths, there are some limitations. First, the authors used the Korean National Elderly Sampled Cohort dataset, which comprises administrative data and not actual medical records, and thus is potentially limited in capturing institutional transition of cancer care. Second, the dataset did not have detailed clinical information such as stage or pharmacologic treatments. However, this study included control variables including CCI, surgery, radiation, and chemotherapy, which may be employed as proxy for the medical status of patients. Moreover, regarding interactions with institutional transition of cancer care, types of treatment at 5-year mortality revealed some significant interactions. Thus, caution must be exercised in evaluating the impact of transition of care on long-term patient outcomes. Third, the dataset in this study might not have captured accurately whether the transition of care was opted for by the patient or recommended by the physician. Fourth, South Korea’s unique insurance and healthcare delivery system may significantly limit the generalizability of the findings of this study to other countries as healthcare utilization depends on the type of health insurance system and the provider’s ability to negotiate the price of healthcare services. Furthermore, it is possible that patient characteristics influenced the results of this study because it was not a randomized controlled or cohort study between the two groups (Transition group vs. Non-transition group). Further studies should be conducted on how this may affect the transition of care delivery and its association with mortality. In addition, the authors investigated only patients with lung cancer. Therefore, the results of this study might differ from outcomes of studies involving patients with other types of cancer, possibly weakening the reliability of the current study’s findings. Moreover, we used the 30 and 90-day-periods from cancer diagnosis for defining the primary visiting hospital based on the healthcare quality assessment criteria of HIRA (surgical treatment rate within 30 days after cancer diagnosis) and the optimal time-to-treatment (27 days) in a previous study that used the same database [27]. However, this could vary depending on the diagnosis-treatment process for each patient and might not fully consider long-term outcomes, as for 5-year mortality, because institutional transition of cancer care was defined based on short-term outcomes. In cancer research, 5-year mortality is commonly used as an indicator of patient outcomes, and it has been considered suitable to compare the appropriate effects of short-term mortality. Nevertheless, the results of this study need to be reviewed carefully. Finally, the data source of this study did not include clinical information details of patients with cancer, which is a limitation of the administrative dataset. The authors used survival time in the model; however, further study using cancer registry data or a cohort dataset, and controlling for proper severity, is warranted. Despite these limitations, to the best of the authors’ knowledge, this is a first attempt to analyze and explore institutional transition of cancer care and its association with mortality in patients with lung cancer.

## Conclusions

This study found evidence that institutional transition of cancer care was associated with higher mortality among elderly lung cancer patients. Patients should be provided with alternatives that optimally guide them through the diagnosis-treatment process and prevent unnecessary institutional transition; currently, there are few mechanisms that regulate this issue in South Korea. Furthermore, health policy makers should be aware of transitional care groups of patients who have a high risk of mortality and need to be monitored.

## Data Availability

Data for this study are public data and can be used through the following NHIS website after application form and fee payment (https://nhiss.nhis.or.kr/bd/ab/bdaba000eng.do).

## Abbreviations

CCI: Charlson Comorbidity Index
HR: Hazard ratio
HIRA: Health Insurance Review and Assessment service
ICD: International Classification of Diseases
NHI: National Health Insurance
